# Aaptamine attenuates the proliferation and progression of non-small cell lung carcinoma

**DOI:** 10.1080/13880209.2020.1822420

**Published:** 2020-10-07

**Authors:** Kaikai Gong, Shuang Miao, Lijuan Yang, Yan Wu, Jiwei Guo, Weiwei Chen, Juanjuan Dai, Jing Du, Sichuan Xi

**Affiliations:** Cancer Research Institute, Binzhou Medical University Hospital, Binzhou, PR China

**Keywords:** NSCLC, CDK, Cylin, PI3K/AKT/GSK3β pathway

## Abstract

**Context:**

Aaptamine is a potent ocean-derived non-traditional drug candidate against human cancers. However, the underlying molecular mechanisms governing aaptamine-mediated repression of lung cancer cells remain largely undefined.

**Objective:**

To examine the inhibitory effect of aaptamine on proliferation and progression of non-small cell lung carcinoma (NSCLC) and dissect the potential mechanisms involved in its anticancer functions.

**Materials and methods:**

*In vitro* assays of cell proliferation, cell cycle analysis, clonal formation, apoptosis and migration were performed to examine the inhibitory effects of aaptamine (8, 16 and 32 μg/mL) on NSCLC cells. The expression levels of proteins were analysed using western blotting analysis when cells were treated with a single drug or a combination treatment for 48 h.

**Results:**

Aaptamine significantly inhibited A549 and H1299 cells proliferation with IC_50_ values of 13.91 and 10.47 μg/mL. At the concentrations of 16 and 32 μg/mL, aaptamine significantly reduced capacities in clonogenicity, enhanced cellular apoptosis and decreased the motile and invasive cellular phenotype. In addition, aaptamine arrested cell cycle at G1 phase via selectively abating cell cycle regulation drivers (CDK2/4 and Cyclin D1/E). Western blotting results showed that aaptamine attenuated the protein expression of MMP-7, MMP-9 and upregulated the expression of cleaved-PARP and cleaved-caspase 3. Moreover, aaptamine inhibited PI3K/AKT/GSK3β signalling cascades through specifically degrading the phosphorylated AKT and GSK3β.

**Discussion and conclusions:**

Aaptamine retarded the proliferation and invasion of NSCLC cells by selectively targeting the pathway PI3K/AKT/GSK3β suggesting it as a potential chemotherapeutic agent for repressing tumorigenesis and progression of NSCLC in humans.

## Introduction

Lung cancer has been notoriously nominated as the number one cancer killer worldwide (Torre et al. 2015; Bray et al. 2018; Herbst et al. 2018; Smith et al. 2018), and still remains the most aggressive malignant tumour with nearly the lowest survival rates (Hirsch et al. 2017; Zugazagoitia et al. 2016). New therapeutic options for this malignant disease are currently under re-evaluation and natural products are promising resources for cancer-targeted therapeutic agents with less side effects and more specific efficacies than those offered by conventional chemotherapeutic drugs (Rayan et al. 2017).

Screening natural compounds for the identification of novel lead compounds with anticancer activity has been highlighted in improving cancer treatments, especially in the search for alternative chemotherapeutic agents to avoid drug-resistance development in current cancer therapies (Mohan et al. 2013; Stuhldreier et al. 2015). Some natural compounds do not only demonstrate a substantial anticancer potency on their own, but also synergize the efficacy of approved anticancer therapeutics if used in combinatorial treatments (Seca and Pinto 2018). These natural compounds arrest human tumorigenesis via differential mechanisms interfering with an ROS formation (Sarwar et al. 2015), abating the cellular microtubular function (Loong and Yeo 2014), or interrupting the nuclear topoisomerases (Wang and Tse-Dinh 2019).

Marine sponges have been considered as an encouraging source of anticancer agents. The marine sponge-derived metabolites have shown efficacy in their anticancer activities and among such metabolites, aaptamine has been identified and evaluated for its potential multiple anticancer activities. This natural molecule has been found to derepress p21 in a p53-independent manner in chronic myeloid leukaemia (CML) cells (Jin et al. 2011) and osteosarcoma cells (Aoki et al. 2006). It also induces G2/M arrest in cell cycle progression of these cancer cells at lower concentrations, and differentially results in apoptosis at higher concentrations (Dyshlovoy et al. 2012). A proteome-based pathway analysis identified the specific interaction of aaptamine with c-myc and p53 in cancer cells (Dyshlovoy et al. 2014). And the intercalation into the DNA may explain its bioactivities such as cytotoxic and antiviral activities (Bowling et al. 2008). Moreover, aaptamine has been recently found to prevent the side effects of the anticancer drug cisplatin in animal studies (Funk et al. 2014).

Nevertheless, the antitumor effect and the underlying mechanisms of aaptamine in lung cancer are still not reported. The PI3K/AKT/GSK3β signal axis and its downstream cell cycle regulation are the most prominent drug targets in cancer treatment. Glycogen synthase kinase 3β (GSK3β) is a serine/threonine kinase playing an important role in growth modulation and tumorigenesis (Chen et al. 2008; Doble and Woodgett 2003; Forde and Dale 2007). The function of GSK3β can be regulated via phosphorylation of GSK3β at Ser9 by several protein kinases such as AKT, MAPK/ERK and PKA in cell survival pathways (Domoto et al. 2016; Walz et al. 2017). PI3K/AKT regulates growth, survival and metabolism in a variety of cells in which AKT is activated through phosphorylation at Ser473 and Thr308, and phosphorylates GSK3β at Ser9 (Hill and Hemmings 2002; Majewska and Szeliga 2017). Cell cycle regulation drivers (CDK2/4 and Cyclin D1/E) play a critical role in G1 progression and their stability can be regulated by GSK3β via posttranslational modifications (Takahashi-Yanaga and Sasaguri 2008).

In this study, we investigated the effects of aaptamine on non-small cell lung carcinoma (NSCLC) cells growth and progression and dissected the mechanism underlying the anticancer functions via specific degradation of the phosphorylated AKT and GSK3β in PI3K/AKT/GSK3β signal axis, which subsequently abated the cell cycle regulation drivers (CDK2/4 and Cyclin D1/E). These results will provide possible therapeutic strategies for lung cancer.

## Materials and methods

### Reagents and chemicals

Aaptamine with a purity of more than 98% was isolated from marine sponge *Aaptos suberitoides* Brøndsted (Suberitidae) as described below. It was dissolved in dimethyl sulphoxide (DMSO) at a concentration of 100 mg/mL and stored at −20 °C. In the experiments, aaptamine was dissolved in culture medium to obtain the desired concentration. Roswell Park Memorial Institute (RPMI)-1640 cell culture medium and foetal bovine serum (FBS) were purchased from Biological Industries (Kibbutz Beit-Haemek, Israel). Penicillin/streptomycin, trypsin, propidium iodide (PI), paraformaldehyde, crystal violet, RNase, Triton X-100, acrylamide/bis (29:1) 30% solution and phenylmethylsulfonyl fluoride (PMSF) were purchased from Sangon (Shanghai, China). Perifosine was purchased from Beyotime (Shanghai, China). Epidermal growth factor (EGF) was purchased from Preprotech (Suzhou, China). Cell counting kit-8 (CCK-8) was purchased from Dojindo (Kumamoto, Kyushu Island, Japan). Annexin V-FITC/PI apoptosis detection kit was purchased from Vazyme (Nanjing, Jiangsu, China). Cell lysis buffer was obtained from the Beyotime Institute of Biotechnology (Shanghai, China). Pierce bicinchoninic acid (BCA) protein assay kit was purchased from Thermo Fisher Scientific (Waltham, MA). Tetramethylethylenediamine (TEMED) was purchased from Biosharp (Hefei, Anhui, China). Chemiluminescent HRP substrate and polyvinylidene difluoride (PVDF) membranes were purchased from Millipore Corporation (Billerica, MA). Antibodies against CDK2 (cat. no. BS9875M), CDK4 (cat. no. BS90281), Cyclin D1 (cat. no. BS1741) and Cyclin E (cat. no. BS90358) were purchased from Bioworld Technology (Nanjing, China). p-CDK4 (cat. no. AF8007) was obtained from Affinity (Changzhou, Jiangsu, China). p-CDK2 was obtained from Bioss (cat. no. BS3483) (Beijing China). p-PI3K (cat. no. B2501), PARP (cat. no. YT6210) and Caspase 3 (cat. no. YT6113) were purchased from Immunoway Biotechnology Company (Plano, TX). α-Tubulin (cat. no. 2125), Cleaved-PARP (cat. no. 9548), Cleaved-caspase 3 (cat. no. 9661), MMP9 (cat. no. 13667), MMP7 (cat. no. 3801), PI3K (cat. no. 4257), AKT (cat. no. 4691), p-AKT (Ser473) (cat. no. 9271), GSK3β (cat. no. 12456), p-GSK3β (cat. no. 5558), horseradish peroxidase (HRP)-conjugated secondary rabbit and mouse antibodies were purchased from Cell Signalling Technology (Beverley, MA).

### Extraction and isolation of aaptamine

The marine sponge *A. suberitoides* was collected from the South Sea (Yongxing Islands sea area) at a depth of 12 m in 2012, and was frozen immediately after collection. The specimen was identified by Dr. Nicole J. de Voogd (National Museum of Natural History, Leiden, the Netherlands). The frozen sample of *A. suberitoides* (2.5 kg, wet weight) was homogenized and then extracted with MeOH three times (5 L × 3, each, 3 d) at room temperature, and the solution was evaporated in vacuum to yield a crude extract (90.2 g) which was subjected to column chromatography (CC) on silica gel using petroleum ether/acetone (from 100:1 to 1:1, v/v) and dichloromethane/methanol (from 20:1 to 0:1, v/v) as eluent to obtain nine fractions (Fr.1–Fr.9). Fr.6 was separated by silica gel CC eluted with dichloromethane/methanol (from 50:1 to 0:1, v/v) to afford eight sub-fractions (Fr.6.1–Fr.6.8). Fr.6.6 was further chromatographed by Sephadex LH-20 eluted with dichloromethane/methanol (1:1, v/v) to yield Fr.6.6.1–Fr.6.6.3. Fr.6.6.2 was further purified by semi-preparative RP-HPLC (C18, MeOH/H2O, 40:60, v/v, 1.5 mL/min) to yield compound aaptamine (62.5 mg). ^1^H-, ^13^C-NMR spectra were recorded on a Varian 500 spectrophotometer. δ in ppm with solvent residual signals as internal standards (DMSO: *δ*H 2.50 ppm, *δ*C 39.5 ppm), *J* in Hz. Spectroscopic data (below) were consistent with data previously reported for aaptamine (Nakamura et al. 1982).

Aaptamine: C_13_H_12_N_2_O_2_, yellow oil, ^1^H NMR (500 MHz, DMSO, TMS) *δ* 12.82 (1H, s, NH-1), 12.30 (1H, s, NH-4), 7.85 (1H, d, *J* = 6.8 Hz, H-2), 7.39 (1H, d, *J* = 6.6 Hz, H-5), 7.10 (1H, s, H-7), 6.86 (1H, d, *J* = 7.0 Hz, H-6), 6.39 (1H, d, *J* = 6.8 Hz, H-3), 3.97 (3H, s, OMe-8), 3.81 (3H, s, OMe-9); ^13^C NMR (DMSO, 125 MHz) *δ* 156.9 (C-8), 149.7 (C-3a), 141.9 (C-2), 133.7 (C-9a), 132.6 (C-6a), 131.4 (C-9), 139.9 (C-5), 116.3 (C-9b), 112.6 (C-6), 100.9 (C-7), 98.1 (C-3), 60.3 (OMe-9), 56.5 (OMe-8).

### Cell cultures

The non-small cell lung cancer cell lines A549 and H1299 were obtained from American Type Culture Collection (ATCC; Manassas, VA). A549 and H1299 cell lines were maintained in RPMI medium supplemented with 10% FBS and 1% penicillin/streptomycin. Cells were incubated at 37 °C with 5% CO_2_.

### Cell viability assay

Cells were plated in 96-well plates at a seeding density of 3000 cells/well. The cells were left to adhere overnight. A549 and H1299 cells treated with aaptamine of 0, 3.125, 6.25, 12.5, 25 and 50 μg/mL for 48 h. Add 10 μL of CCK-8 solution to each well of the plate and then incubate the plate for 4 h in the incubator. Measure the absorbance at 450 and 630 nm using a microplate reader (Bio-Rad, Fitchburg, WI). The half-maximal inhibitory concentration (IC_50_) of aaptamine in A549 and H1299 cells was calculated using Graphpad Prism version 7 (GraphPad Software, La Jolla, CA). In the cell proliferation analysis, CCK-8 test was performed 12, 24, 36, 48, 60 and 72 h after A549 and H1299 cells treatment with various concentrations (0, 8, 16 and 32 μg/mL) of aaptamine. Cell viability was calculated as the relative absorbance compared to control absorbance. All experiments were repeated three times in three wells of the microplate. All experiments were repeated three times in three wells of the microplate (Chen et al. 2018).

### Cell cycle assay

Cells were counted and seeded into 12-well plate with 500 cells per well and cultured for 24 h before drug incubation. Cells were treated with indicated concentrations (0, 8, 16 and 32 μg/mL) of aaptamine for 48 h and then cultured in drug-free medium for another 10–12 d. Cell clones were fixed with freshly prepared 4% paraformaldehyde and stained with crystal violet before optical imaging. Experiments were carried out in triplicates and the number of colonies was counted for analysis (Shrestha et al. 2018).

### Cell apoptosis assay

Cells treated with various concentrations (0, 8, 16 and 32 μg/mL) of aaptamine for 48 h were collected, washed with pre-cold PBS and stained with Annexin V/PI mixture at 4 °C for 30 min according to the manufacturer’s instructions (Vazyme, China). After full resuspension, fluorescent signals were measured with CFlow Plus package from Accuri C6. To form four-quadrant dot plots, x- and y-axis were set up as FL1 and FL2 channel separately. Dots in left lower quadrant represented viable cells. Dots in right lower and upper quadrants indicated early apoptotic and terminal apoptotic cells, respectively. Data were analysed using FlowJo version 7.6.1 software (FlowJo LLC, Ashland, OR, USA) (Bian et al. 2019).

### Wound healing assay

Cells were seeded into 12-well plate at 10^5^ cells per well 24 h before treatment. On the second day, cells were scraped with a 200 μL tip followed by incubation with serum-free RPMI medium containing 0, 8, 16 and 32 μg/mL aaptamine for 24 or 48 h. Photomicrographs were taken using Olympus light microscope IX 53 (Tokyo, Japan) of a same area before and 24 or 48 h after treatment. The remaining wound area was quantified with ImageJ software. The migration rate = (Wound width at the beginning of experiment − Wound width at the end of experiment for 24 or 48 h)/Wound width at the beginning of experiment (Zeng et al. 2019).

### Transwell assay

Cells were treated with various concentrations (0, 8, 16 and 32 μg/mL) of aaptamine for 48 h. After that, 3000 of the treated cells were seeded into RPMI without FBS in the upper chamber. RPMI (600 μL) containing 20% FBS was added to the lower chamber. The cells were allowed to migrate for 60 h at 37 °C in a humidified atmosphere containing 5% CO_2_. Cells remaining on the upper side of the membrane were removed using PBS-soaked cotton swabs. The membrane was then fixed in 4% paraformaldehyde for 20 min at 37 °C and then stained with crystal violet. Cells on the lower side of the membrane were counted under an Olympus light microscope (Olympus, Tokyo, Japan) at ×100 magnification (Zeng et al. 2019).

### Reverse transcriptase quantitative real-time PCR (qRT-PCR)

Total RNA was extracted with the Trizol reagent (TransGen Biotech, Beijing, China) from the cells. The TransScript All-in-One First-Strand cDNA Synthesis Kit (TransGen Biotech) was used to synthesize cDNA, and qRT-PCR was conducted using the 2 × SYBR Green Master Mix (Qiagen, Germany). The relative normalized expression of the target genes was compared with that of the control gene, and the mRNA expression of each gene was calculated with the 2^−ΔΔCt^ method (Chen et al. 2018). The primers are as follows:

Cyclin D1 forward primer: 5′-AGGTGCTTCTGCTGTGC-3′
Cyclin D1 reverse primer: 5′-CCTTCTTCCTCCCTCACTTCTC-3′
Cyclin E forward primer: 5′-CCACCTCCATVACCACTACCA-3′
Cyclin E reverse primer: 5′-CCATTCCAACCAGAGCCACC-3′
CDK2 forward primer: 5′-AACACAGAGGGGGCCATCAAGC-3′
CDK2 reverse primer: 5′-CAGGAGCTCGGTACCACAGGGTC-3′
CDK4 forward primer: 5′-ACCAGATGGCACTTACACCC-3′
CDK4 reverse primer: 5′-TCCACAGAAGAGAGGCTTTCG-3′

### Western blot analysis

Cells were harvested and lysed on ice for 30 min in ice-cold lysis buffer containing protease and phosphatase inhibitor cocktail. The protein concentration was determined by BCA protein assay kit. Equivalent amounts of proteins were separated on sodium dodecyl sulphate-polyacrylamide gels (SDS-PAGE) and transferred to PVDF membranes by electroblotting. After blocking with 5% skim milk, the membranes were incubated with the respective primary antibody (1:1000 dilutions) at 4 °C overnight, followed by HRP-conjugated secondary antibodies. Finally, the immunoreaction was visualized using ECL HRP substrate and detected using Bio-rad ChemiDoc XRS + system (Hercules, CA). The band intensity was quantified using ImageJ software (Zhu et al. 2020).

### Statistical analysis

Data were analysed with Graphpad Prism version 7 software and results were presented as the means ± SD. One-way ANOVA with a Tukey’s *post hoc* test was used to compare multiple groups. A *p* value < 0.05 was considered statistically significant and marked by an asterisk. All quantifications were performed with at least three independent experiments. A *p* value < 0.01 was marked by two asterisks. A *p* value < 0.001 was marked by three asterisks.

## Results

### Aaptamine decelerates cell proliferation, invasive growth and clonogenicity of NSCLC cells

**Figure 1.**
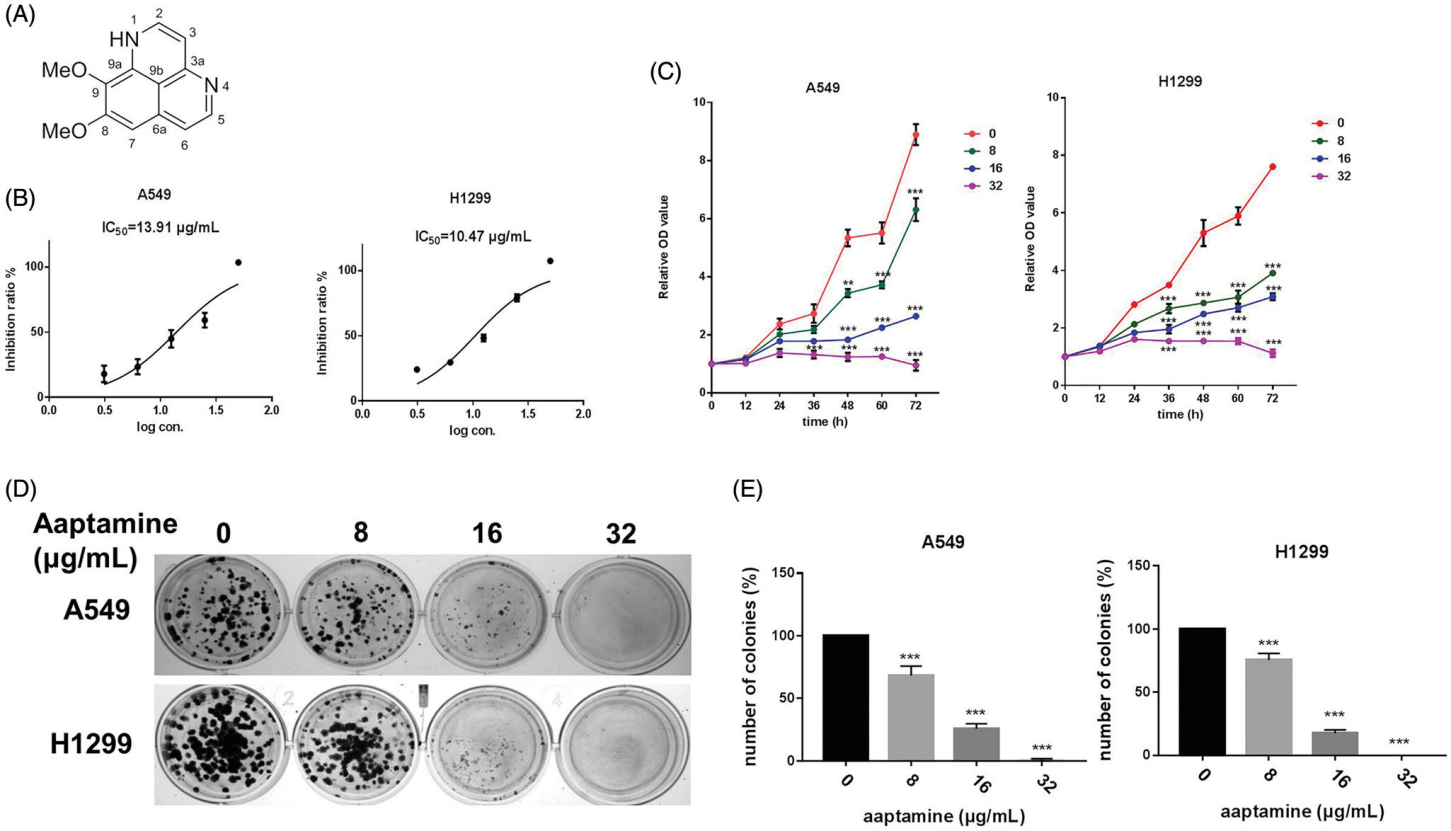
Aaptamine arrests NSCLC cell growth. (A) Chemical structure of aaptamine, the isolated bioactive compound from sponge *Aaptos suberitoides.* (B) Cell proliferation assay of A549 and H1299 cells treated with different concentrations of aaptamine (0, 3.125, 6.25, 12.5, 25 and 50 μg/mL). Each experiment was performed in triplicate. Results were plotted in Graphpad Prism version 7; Bars represent mean ± SD. (C) CCK-8 assay of A549 and H1299 treated with different concentrations of aaptamine (0, 8, 16 and 32 μg/mL) for 12, 24, 36, 48, 60 and 72 h before CCK-8 test. The OD values for all concentrations were normalized to that of corresponding control cells. (D) Colonal formation assay of A549 and H1299 after treatment of different concentrations of aaptamine (0, 8, 16 and 32 μg/mL) for 10–12 d. (E) Quantitative representation of the reduction in number of colonies was shown by histogram. Bars represent mean ± SD. *p < 0.05, **p < 0.01 and ***p < 0.001 *vs.* untreated control.

**Figure 2.**
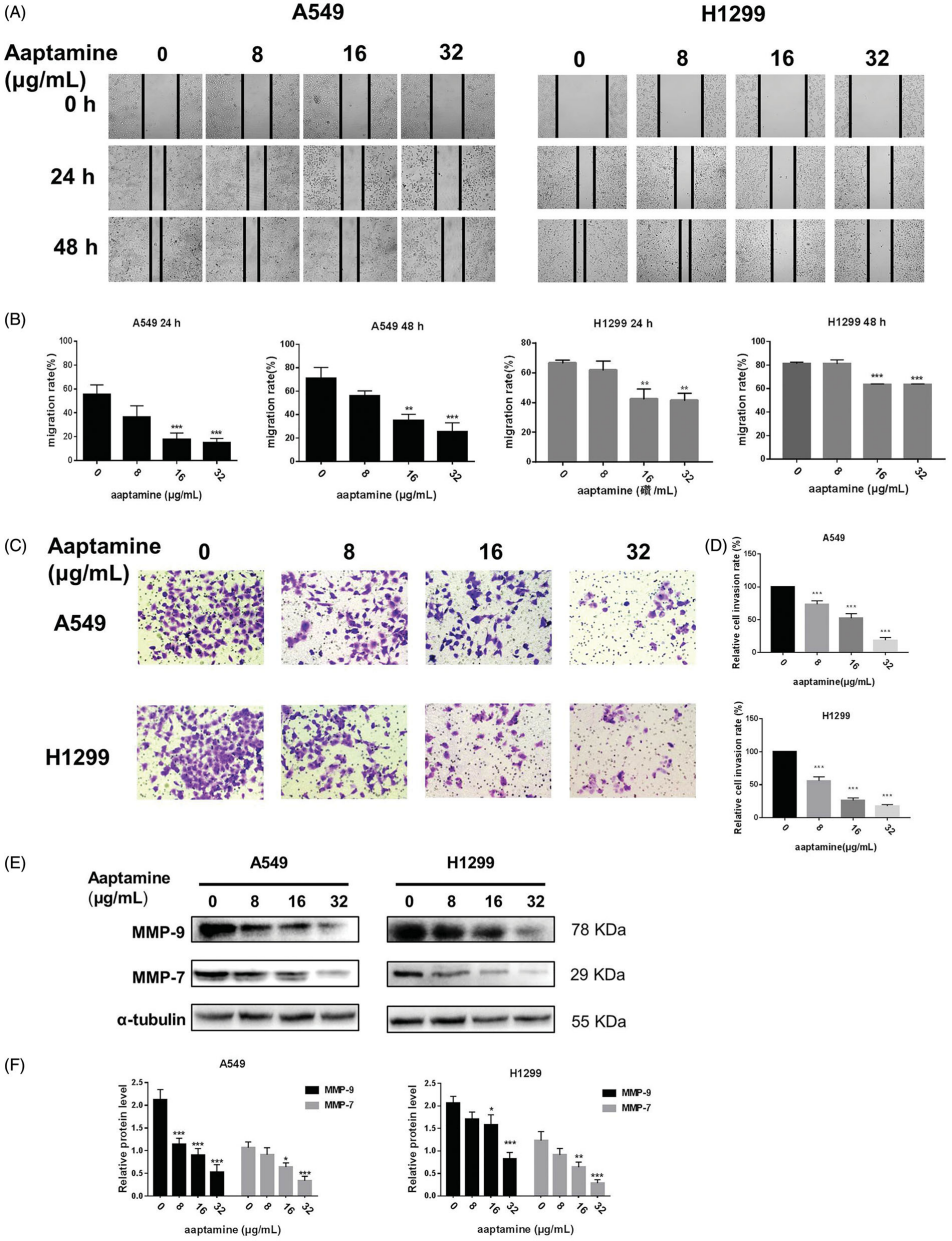
Aaptamine represses invasive growth of NSCLC cells. (A) The wound-healing assay showed representative effects of aaptamine (0, 8, 16 and 32 μg/mL) on A549 and H1299 cell migration at 24 and 48 h both representatively and (B) quantitatively. (C-D) Similar results were obtained in transwell assays, where aaptamine decreased the number of invading cells. (E,F) Western blot assay of A549 and H1299 cells treated with aaptamine (0, 8, 16 and 32 μg/mL), respectively, for 48 h demonstrated that aaptamine decreased the protein level of MMP-7 and MMP-9. Scale bars represent 100 μm. Bars represent mean ± SD. *p < 0.05, **p < 0.01 and ***p < 0.001 *vs.* untreated control.

Aaptamine, a bioactive compound isolated from the sponge *A. suberitoides* (Figure 1(A)), is a potent ocean-derived non-traditional drug candidate against human cancers. To investigate the potential biological function of aaptamine in lung cancer, we first examined whether aaptamine arrests the NSCLC cell growth. Cell proliferation assays of two tumour cells A549 and H129 treated with different concentrations of aaptamine for 48 h defined the 50% cell growth inhibitory capacities of aaptamine more specifically and significantly for lung tumour cells compared to that in the normal lung cells (Figure 1(B)). Additional CCK-8 assay detection displayed that aaptamine repressed A549 and H1299 cell proliferation in a time and dose-dependent manner when administered at various concentrations (8, 16 and 32 μg/mL) (Figure 1(C)). Colonal formation assay revealed that aaptamine opposed the clonogenecity of A549 and H1299 cells after treatment of different concentrations of aaptamine (8, 16 and 32 μg/mL) for 10–12 d (Figure 1(D,E)). To disclose anti-metastatic effect of NSCLC cells by aaptamine, the wound-healing assay (Figure 2(A,B)) and tranwell assay (Figure 2(C,D)) showed representative effects of aaptamine (8, 16 and 32 μg/mL) on A549 and H1299 cells. Consistently, the related protein involved in cellular migration and invasion, MMP9 and MMP7 expression level was decreased significantly and dose-dependently by aaptamine treatment (Figure 2(E,F)).

### Aaptamine suppresses cellular viability *via* induction of apoptosis in NSCLC cells

**Figure 3.**
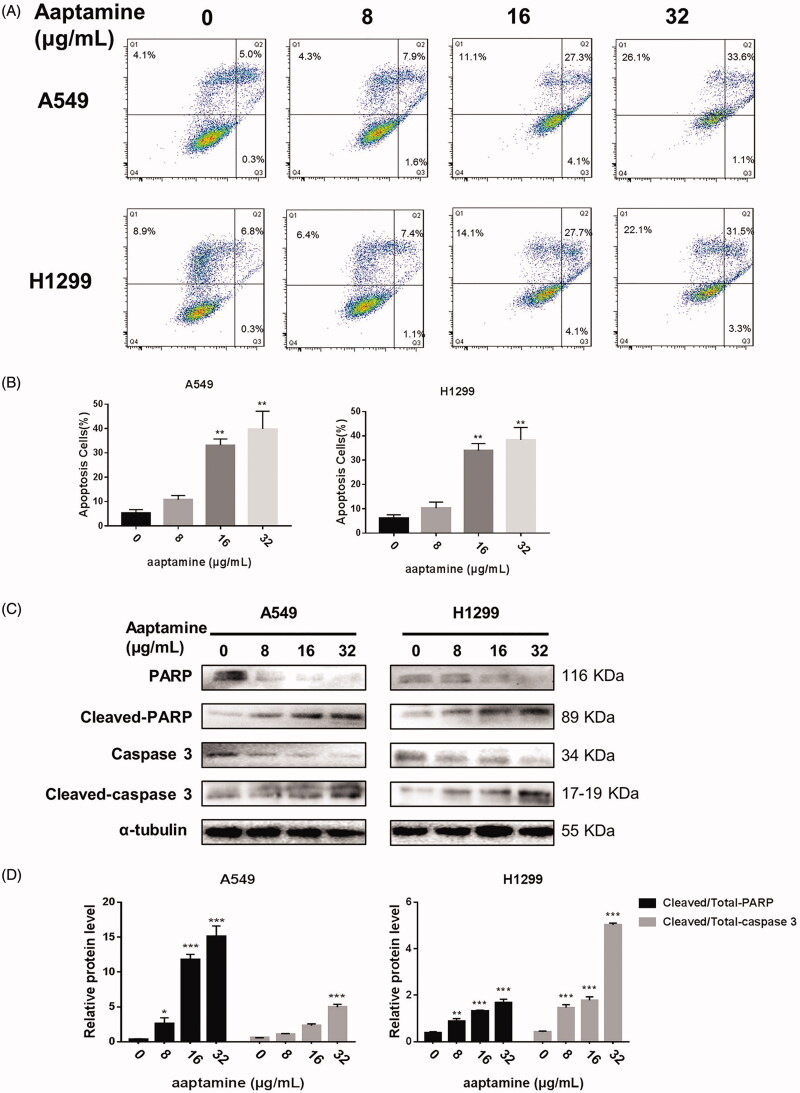
Aaptamine enhances apoptotic cell death of NSCLC cells. (A) A549 and H1299 cells were incubated with different concentrations of aaptamine (0, 8, 16 and 32 μg/mL) for 48 h and subjected to apoptosis tests. (B) Proportion of early apoptotic and late apoptotic cells were analysed. (C,D) Western blot assay of A549 and H1299 cells treated with aaptamine (0, 8, 16 and 32 μg/mL), respectively, for 48 h demonstrated that aaptamine increased the protein level of cleaved-PARP and cleaved caspase-3 and decreased those of Total-PARP and Total-caspase-3. Bars represent mean ± SD. *p < 0.05, **p < 0.01 and ***p < 0.001 *vs.* untreated control.

Cellular apoptosis resistance is the death-avoiding mechanism of tumour cells, and serves as one of the chief therapeutic targets. To examine whether aaptamine induced apoptosis in the NSCLC cells, A549 and H1299 cells were incubated with different concentrations of aaptamine (0, 8, 16 and 32 μg/mL) for 48 h. Apoptosis tests demonstrated that the proportion of early apoptotic and late apoptotic cells were significantly elevated at the concentrations of 16 and 32 μg/mL (Figure 3(A,B)). Next, we examined the expression of the apoptotic markers in NSCLC cells exposed to aaptamine. Accordingly, augmented expression of cleaved form of PARP and caspase 3 was detected in aaptamine-treated NSCLC cells (Figure 3(C,D)).

### Aaptamine blocks cell cycle progression through attenuating cell cycle-specific driver kinases and proteins in NSCLC cells

**Figure 4.**
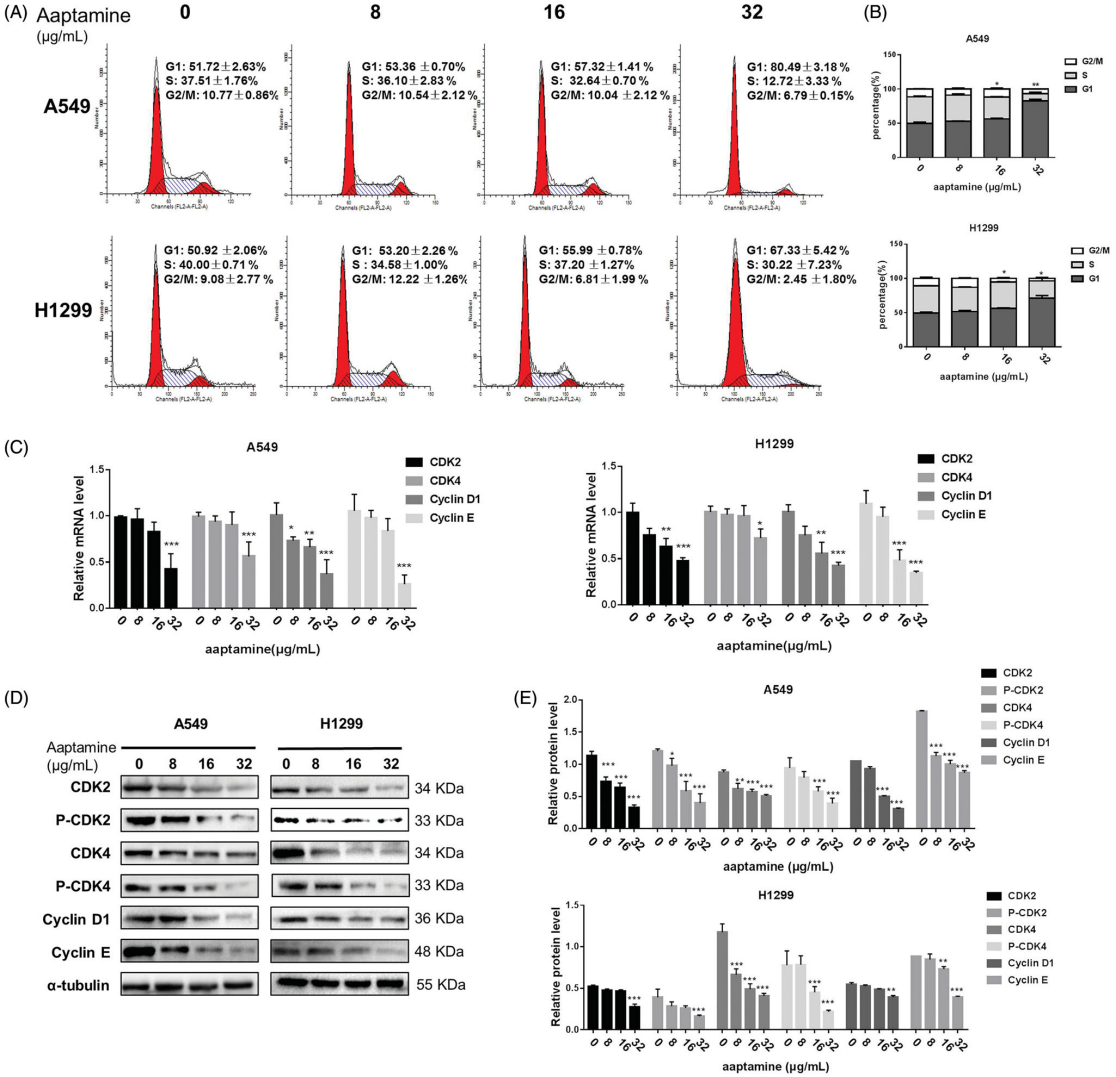
Aaptamine balks cell cycle progression of NSCLC cells. (A,B) Cell cycle analysis of A549 and H1299 cells treated with different concentrations of aaptamine (0, 8, 16 and 32 μg/mL) for 48 h profiling the cell cycle distribution by ModFit LT 3.3. Each experiment was performed in triplicate. The percentage of cells in each phase of the cycle is shown as the mean ± SD and represented as bar diagram. (C–E) Aaptamine attenuates cell cycle-specific driver kinases and proteins in NSCLC cells. RT-qPCR analysis (C) and immunoblotting assay (D and E) of A549 and H1299 cells treated with aaptamine (0, 8, 16 and 32 μg/mL), respectively, for 48 h with densitometric analysis showing that aaptamine could decrease the mRNA and protein level of cell cycle-specific driver kinases in a dose-dependent manner. *p < 0.05, **p < 0.01 and ***p < 0.001 *vs.* untreated control.

Cell cycle progression interference is the most common anti-tumour mechanism of the currently available chemotherapeutic agents. To dissect the functional relevance of aaptamine in lung cancer, herein we examined the cell cycle arrest in lung cancer cells after exposure to aaptamine and found that aaptamine balked cell cycle progression of NSCLC cells. Treatment of A549 and H1299 cells with different concentrations of aaptamine (16 and 32 μg/mL) for 48 h induced significant G1 arrests and S reduction as demonstrated by the representative histograms of cell cycle distribution and their quantitation analysis (Figure 4(A,B)). Subsequently, profiling the expression of cell cycle-specific driver kinases and proteins revealed that aaptamine attenuates cell cycle-specific driver kinases and proteins in NSCLC cells. Both RT-qPCR (Figure 4(C)) and immunoblotting assay (Figure 4(D,E)) of A549 and H1299 cells treated with aaptamine (0, 8, 16 and 32 μg/mL), respectively, for 48 h with densitometric analysis demonstrated that aaptamine could decrease the RNA and protein level of CDK2, CDK4, Cyclin D1 and Cyclin E in a dose-dependent manner.

### Aaptamine deactivates PI3K/AKT/GSK3β axis and downstream disrupts the cell cycle maintenance in NSCLC cells

**Figure 5.**
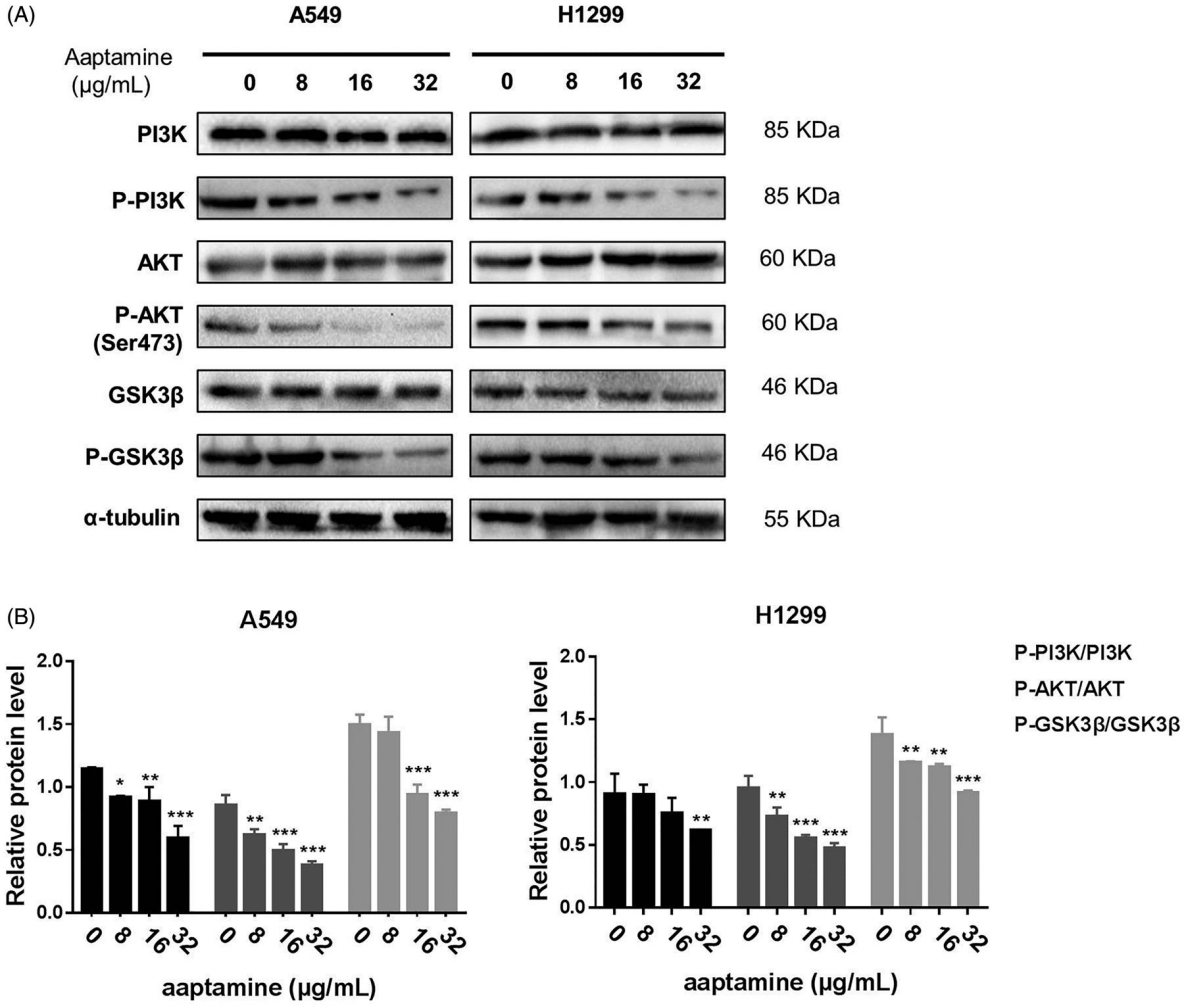
Aaptamine deactivates PI3K/AKT/GSK3β in NSCLC cells. (A) Western blot assay of A549 and H1299 cells treated with aaptamine (0, 8, 16 and 32 μg/mL), respectively, for 48 h with densitometric quantitation (B) demonstrating that aaptamine dephosphorylated both AKT and GSK3β to lower the levels of p-AKT and p-GSK3β in a dose-dependent manner of NSCLC cells. Bars represent mean ± SD. *p < 0.05, **p < 0.01 and ***p < 0.001 *vs.* untreated control.

**Figure 6.**
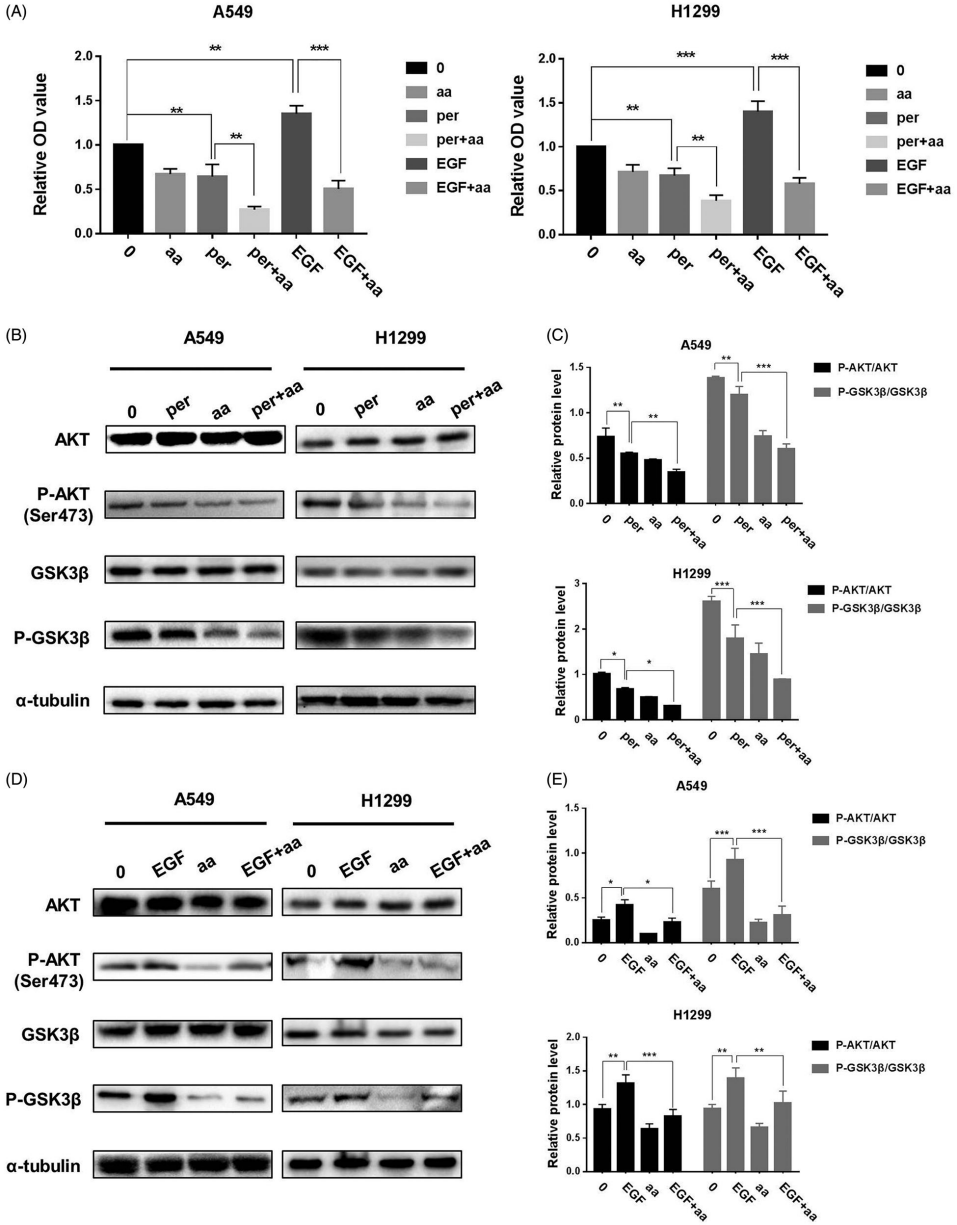
The activation of PI3K/AKT/GSK3β pathway offsets the antitumor activity of aaptamine. (A) CCK-8 assay of A549 and H1299 cells treated with aaptamine (32 μg/mL), perifosine (20 μM), EGF (100 ng/mL) alone or in combination for 48 h. The OD values for all concentrations were normalized to that of corresponding control cells. (B) Immunoblotting assay of A549 and H1299 cells treated with perifosine (20 μM), aaptamine (32 μg/mL) or both for 48 h with densitometric quantitation (C). *p < 0.05, **p < 0.01 and ***p < 0.001 *vs.* perifosine treated groups. (D) Immunoblotting assay of A549 and H1299 cells treated with EGF (100 ng/mL), aaptamine (32 μg/mL) or both for 48 h with densitometric quantitation (E). *p < 0.05, **p < 0.01 and ***p < 0.001 *vs.* EGF treated groups. Bars represent mean ± SD.

Cell cycle progression is modulated by varieties of upstream signalling cascades in NSCLC cells. Aberrant activation of PI3K/AKT/GSK3β axis is one of the critical initial biological events in deregulating cell cycle rhythm in lung cancer cells. To interrogate whether aaptamine-induced deactivation of PI3K/AKT/GSK3β axis disrupted the cell cycle progression of NSCLC cells, western blot assay of A549 and H1299 cells treated with aaptamine (0, 8, 16 and 32 μg/mL), respectively, for 48 h illuminated that aaptamine specifically decreased the ratios of p-PI3K/PI3K, p-AKT/AKT and p-GSK3β/GSK3β in a dose-dependent manner, which correspondingly downregulated their downstream cell cycle-specific driver kinases and proteins in NSCLC cells (Figure 5). To further validate whether the aaptamine-induced cell cycle arrest in lung cancer cells was due to specific dephosphorylation of both AKT and GSK3β, AKT antagonist perifosine and its agonist EGF were applied. Perifosine is an alkyphospholipid that targets the pleckstin homology domain of AKT and blocks its membrane translocation, hence preventing AKT phosphorylation and activation (Becher et al. 2010). CCK8 assay showed that co-treatment of aaptamine (32 μg/mL) with perifosine (20 μM) significantly and synergistically blocked proliferation of A549 and H1299 cells. On the contrary, the pro-proliferative effect of EGF on NSCLC cells was significantly reversed by co-treatment with aaptamine (Figure 6(A)). Protein level detection using immunoblot assay also agrees with our data as demonstrated by CCK-8. Aaptamine (32 μg/mL) synergized perifosine (20 μM)-mediated dephosphorylation while it counteracted EGF (100 ng/mL) induced activation of both AKT and GSK3β in NSCLC cells (Figure 6(B–E)). Therefore, aaptamine selectively interfered with PI3K/AKT/GSK3β axis to disrupt cell cycle maintenance and growth of NSCLC cells.

## Discussion

In this study, we explored the oncogenic signalling pathway-specific bioactive mechanisms, and evaluated the specificity and efficacy of the compound aaptamine derived from the marine sponge, in arresting the NSCLC progression. Our study revealed that aaptamine blocked cell cycle progression to repress cell growth, to diminish clonogenicity and to attenuate cellular viability and invasive growth of NSCLC cells in which aaptamine dephosphorylated AKT and GSK3β in PI3K/AKT/GSK3β signalling cascades and targeted downstream cell cycle regulation drivers (CDK2/4 and Cyclin D1/E). Aaptamine is a potential therapeutic drug candidate in targeting cell cycle-related signalling pathways for NSCLC.

Currently available therapeutic options and advancements in lung cancer earlier diagnosis have improved the survival of patients substantially over the past decades, though lung cancer globally remains one of the most aggressive malignancies (Romaszko and Doboszyńska 2018). Clinically, palliative treatments such as chemotherapy and radiotherapy remain the limited choices for lung cancer patients with advanced and unresectable disease (Tomasetti et al. 2017). Moreover, these aggressive treatment selections are unable to be tumour-specific enough without affecting non-tumour cells or tissues (Mun et al. 2018). Therefore, to screen and identify those novel natural compounds with less toxicity and more specific potency in blocking tumour progression is highly demanded in lung cancer therapy.

Aaptamine interferes with tumour progression via application multiple mechanisms such as activation of the tumour suppressor p21, induction of cell cycle arrests and cellular death and binding to the target oncogene c-myc in cancer cells (Aoki et al. 2006; Tsukamoto et al. 2010; Jin et al. 2011; Stuhldreier et al. 2015). This study functionally confirmed that aaptamine disrupted cell cycle progression which phenotypically and specifically inhibited tumour cell proliferation, repressed acquisition of clonogenicity and reduced cell death evasive ability and invasive growth of NSCLC cells *in vitro*.

Cell cycle regulation and its upstream PI3K/AKT/GSK3β signal axis are the most distinguished drug targets in cancer therapies among which phosphorylation of GSK3β and AKT is pivotal in regulating cell growth, survival and metabolism in a variety of cells through stabilizing cell cycle regulation driver proteins (CDK2/4 and Cyclin D1/E) (Chen et al. 2008; Kim et al. 2018). Our results demonstrated that aaptamine significantly and selectively dephosphorylated AKT and GSK3β in PI3K/AKT/GSK3β signalling cascades and concomitantly decreased cell cycle regulation drivers (CDK2/4 and Cyclin D1/E) both transcriptionally and post-transcriptionally. Besides, aaptamine enhanced the inhibitory effect of AKT antagonist perifosine. These experiments illustrated the mechanism by which aaptamine interacted with specific signalling molecules and their signalling networks in lung cancer cells in details which provided the first evidence to characterize aaptamine as a potential therapeutic drug candidate in targeting cell cycle-related signalling pathways for NSCLC.

The specificity of any anticancer drug is one of the most critical and significant issues to be considered in both preclinical and clinical contexts (Kashyap et al. 2019). Our *in vitro* assays demonstrated that aaptamine arrested the cellular proliferation and the invasive growth of lung tumour cells (A549 and H1299), which preliminarily revealed the potential of aaptamine as a novel cancer antagonist with significant and selective inhibition of cancer cells. The mechanism underlying the specificity in the aaptamine-induced anticancer function is the regulation of cell cycle and its upstream PI3K/AKT/GSK3β signal axis in which aaptamine is sensitive to dephosphorylate AKT and GSK3β in lung cancer cells.

Aaptamine displayed promising bioactivities against lung cancer in this study which does not camouflage the research limitations in comprehensively evaluating the sensitivity and specificity of its anticancer functions. There remain some major issues to be addressed, which at least extends our experiments to explore whether aaptamine effectively alleviates the unavoidable chemotherapeutic refractory development of routine lung cancer treatment when applied in combination with other lung cancer treatments and whether its application significantly prevents *in vivo* metastasis in lung cancer. In our future studies, the mechanisms governing these phenotypic functions will need to be examined further. Additionally, aaptamine has shown cytotoxic activities against tumour cells via its intercalation into the DNA and meanwhile it prevented the side effects of the anticancer drug cisplatin in animal studies which suggest aaptamine may function in context-dependent manners (Dyshlovoy et al. 2014). In summary, aaptamine selectively represses cell cycle regulation drivers (CDK2/4 and Cyclin D1/E) and the upstream regulator cascades (PI3K/AKT/GSK3β) to retard the cell proliferation and invasive growth of NSCLC cells. Our results suggest aaptamine as a potential chemotherapeutic agent for repressing tumorigenesis and progression of NSCLC in humans, which grants any further clinical investigation since aaptamine potently and specifically targets cell cycle-related signalling pathways.
